# Integrative skin–blood transcriptomic analysis identifies circulating biomarkers reflecting disease activity in atopic dermatitis

**DOI:** 10.3389/falgy.2026.1837776

**Published:** 2026-06-09

**Authors:** Alberto Fernández-Bernaldez, Ana Jiménez-Sánchez, Pablo Chicharro, Celia González-Agudo, Fátima Sánchez-Cabo, Alicia Vara, Mar Llamas-Velasco, Manuel José Gómez, Hortensia de la Fuente

**Affiliations:** 1 Dermatology Department, Instituto de Investigación Sanitaria Hospital Universitario de La Princesa IIS-Princesa, Madrid, Spain; 2 Immunology Department, Instituto de Investigación Sanitaria Hospital Universitario de La Princesa IIS-Princesa, Madrid, Spain; 3 Bioinformatics Unit, Centro Nacional de Investigaciones Cardiovasculares, CNIC-ISCIII, Madrid, Spain; 4 Centro de Investigación Biomédica en Red Enfermedades Cardiovasculares (CIBERCV), Madrid, Spain

**Keywords:** atopic dermatitis, disease activity, immune regulation, peripheral blood biomarkers, transcriptomics

## Abstract

**Background.:**

Atopic dermatitis (AD) is characterized by complex immune dysregulation primarily studied in lesional skin. Identifying circulating molecular signatures that mirror cutaneous inflammation could enable non-invasive disease monitoring.

**Methods.:**

Transcriptomic data from lesional skin and peripheral blood T lymphocytes of patients with AD were analyzed using differential expression, pathway enrichment, upstream regulator prediction, and protein–protein interaction network modeling. Genes dysregulated in both compartments were prioritized and validated in serum samples from patients with moderate-to-severe AD.

**Results.:**

Despite limited concordance at the individual gene level, skin and blood shared a common inflammatory regulatory landscape, including activation of Th2- and Th17-related cytokine signaling, together with a consistently prediction of ESR1 inhibition across both compartments. Network analysis identified key hub genes and a neuro-immune signaling module. Among shared molecules, serum S100A8 levels positively correlated with disease severity, while SOCS3 showed a negative association. LCN2 and CTLA4 displayed trends toward correlation.

**Conclusions.:**

Systemic molecular alterations in AD partially reflect cutaneous inflammation and support circulating S100A8 and SOCS3 as potential biomarkers of disease activity.

## Introduction

Atopic dermatitis (AD) is a chronic inflammatory skin disease characterized by pruritic, relapsing eczematous lesions and a highly heterogeneous clinical presentation. Although it often begins in childhood, AD frequently persists into adulthood and is associated with a substantial disease burden, including allergic comorbidities and impaired quality of life (1). From an immunological perspective, AD has classically been considered a Th2-driven disease; however, increasing evidence supports the contribution of additional immune pathways, including Th17, Th22 and Th1 responses, which vary according to disease stage, severity and patient endotype (2).

Advances in transcriptomic technologies have substantially improved our understanding of AD pathogenesis by enabling the comprehensive characterization of molecular alterations within lesional skin (3). These studies have revealed complex inflammatory programs involving cytokine signaling, barrier dysfunction or lipid metabolism. In contrast, gene expression studies in peripheral blood cells from patients with AD remain comparatively limited (4, 5). This imbalance restricts the identification of circulating molecular signatures that could serve as accessible biomarkers for disease monitoring or provide insight into the systemic component of the disease.

Whether molecular changes occurring in AD skin are reflected in peripheral blood remains incompletely understood. Previous studies suggest that blood-based signatures may capture aspects of immune activation and cardiovascular or inflammatory risk (6); however, direct integrative analyses comparing skin and peripheral immune compartments are scarce and often yield limited overlap at the level of individual differentially expressed genes (7, 8). This raises the possibility that shared disease mechanisms may be more readily detected at the level of regulatory pathways or molecular networks rather than isolated genes.

In this study, we performed an integrative transcriptomic analysis of lesional skin and peripheral blood T lymphocytes from patients with AD using publicly available datasets. By combining differential gene expression, pathway enrichment, upstream regulator prediction and protein–protein interaction network analyses, we aimed to identify molecular programs shared between skin and blood. We further sought to determine whether selected candidate molecules dysregulated in both compartments could be detected in serum and correlate with disease severity, thereby providing potential biomarkers for clinical monitoring in atopic dermatitis.

## Methods

### Datasets

Publicly available transcriptomic datasets were retrieved from the Gene Expression Omnibus (GEO) (http://www.ncbi.nlm.nih.gov/geo/). The dataset GSE121212 comprises RNA sequencing data from lesional skin biopsies obtained from 21 patients with atopic dermatitis and 38 healthy controls (9). Gene expression data from peripheral blood T lymphocytes were obtained from dataset GSE124701, which includes array-based transcriptomic profiles generated from pooled samples of peripheral blood T cells from 16 patients with atopic dermatitis and 10 healthy controls (7).

### Gene expression analysis

RNA-seq data from the GSE121212 dataset were processed using a standardized bioinformatics pipeline. Quality control of raw sequencing reads was performed using FastQC, followed by adapter trimming with Cutadapt (10). Processed reads were aligned to the Homo sapiens GRCh38.104 reference transcriptome using RSEM to obtain gene-level expression estimates. Differential gene expression analysis comparing atopic dermatitis skin samples with healthy controls was conducted using the *limma* package. *P* values were adjusted for multiple testing using the Benjamini–Hochberg method, and genes with an adjusted *p* value <0.05 were considered significantly differentially expressed. For downstream analyses, differentially expressed genes were further filtered using an absolute log fold change threshold (|logFC| > 1).

Gene expression data from peripheral blood T lymphocytes (GSE124701) were log-transformed and quantile-normalized prior to analysis. As this dataset was generated from pooled samples, statistical testing for differential expression was not feasible. Therefore, gene expression changes were described based on fold-change values, and differentially expressed genes were defined using an absolute log fold change threshold (|logFC| > 1).

### Protein–protein interaction network analysis

Protein–protein interaction (PPI) networks were constructed from the sets of differentially expressed genes using the STRING database. Networks were visualized and analyzed using Cytoscape (version 3.9). Hub genes were identified based on betweenness centrality, a network metric that reflects the relative importance of nodes in information flow across the network.

Clustering analysis was performed using the Markov Cluster Algorithm (MCL) implemented in the ClusterMaker plugin to identify functional modules within the networks. Enrichment of Gene Ontology biological processes within each cluster was assessed using the Biological Networks Gene Ontology (BiNGO) plugin, applying a false discovery rate (FDR) threshold of <0.05.

### Functional enrichment and upstream regulator analyses

Functional enrichment analyses were performed using Ingenuity Pathway Analysis (IPA) and Gene Set Enrichment Analysis (GSEA). IPA was used to identify enriched canonical pathways and to predict upstream regulators based on differential gene expression patterns, providing activation *z*-scores that reflect the predicted direction of regulator activity.

GSEA was conducted using ranked gene lists against the Gene Ontology Biological Process and Immunological Signature gene set collections from the Molecular Signatures Database (MSigDB) (11). Gene sets with a false discovery rate (FDR) <0.05 were considered significantly enriched.

### Validation cohort

Serum samples from 15 patients with moderate-to-severe atopic dermatitis were used for validation analyses. Samples were collected prior to initiation of systemic treatment and were obtained from a registered biobank collection (C.0005113). All participants provided written informed consent, and the study protocol was approved by the Clinical Research Ethics Committee of Hospital Universitario de La Princesa.

### Serum protein analysis

Serum levels of CD83, CTLA4, CXCL8, S100A8 and LCN2 were quantified using a customized multiplex antibody array according to the manufacturer's instructions. Serum levels of SOCS3 and S100A9 were measured using commercially available enzyme-linked immunosorbent assay (ELISA) kits. Correlations between serum protein levels and disease severity, assessed by the Eczema Area and Severity Index (EASI), were evaluated using Spearman's rank correlation coefficient.

### Statistical analysis

Statistical analyses were performed using GraphPad Prism (version 10.4.2) and RStudio (version 2024.12.1). Correlations between serum molecule levels and disease severity (EASI score) were assessed using Spearman's rank correlation coefficient in GraphPad Prism. Receiver operating characteristic (ROC) curve analyses were performed in RStudio to evaluate the ability of individual biomarkers to discriminate disease severity, using a predefined EASI threshold (>21). The area under the curve (AUC) and corresponding 95% confidence intervals (CI) were calculated. A *p*-value < 0.05 was considered statistically significant.

## Results

### Differential gene expression in skin and peripheral blood T cells

Re-analysis of the RNA-seq dataset from lesional skin biopsies (GSE121212) identified 10,888 differentially expressed genes (DEGs) in patients with atopic dermatitis compared with healthy controls (adjusted *p* < 0.05). Of these, 2,180 genes showed an absolute log fold change (logFC) greater than 1, including 1,052 upregulated and 1,128 downregulated genes (Supplementary Table 1).

Analysis of gene expression data from peripheral blood T lymphocytes (GSE124701) identified 282 DEGs with absolute logFC > 1 when comparing AD patients with healthy controls, comprising 152 upregulated and 130 downregulated genes (Supplementary Table 2). As this dataset was generated from pooled samples, differential expression was assessed based on fold-change thresholds rather than statistical significance.

Overall, the magnitude and number of transcriptional alterations were substantially greater in lesional skin than in peripheral blood T cells, reflecting the tissue-specific nature of inflammation in atopic dermatitis.

### Skin and peripheral T cells share common inflammatory regulatory programs

To explore whether skin and peripheral blood T cells share common molecular drivers despite limited overlap at the level of individual genes, functional enrichment and upstream regulator analyses were performed. Ingenuity Pathway Analysis (IPA) of skin DEGs confirmed the activation of well-established inflammatory pathways in atopic dermatitis, including Th2 signaling, IL-17 signaling, Th1 pathway and T cell receptor signaling. In addition, pathways related to neurotransmitter metabolism and neuro-immune communication were identified, while pathways associated with matrix remodeling and antioxidant responses were predicted to be inhibited (Supplementary Table 3).

**Figure 1:**
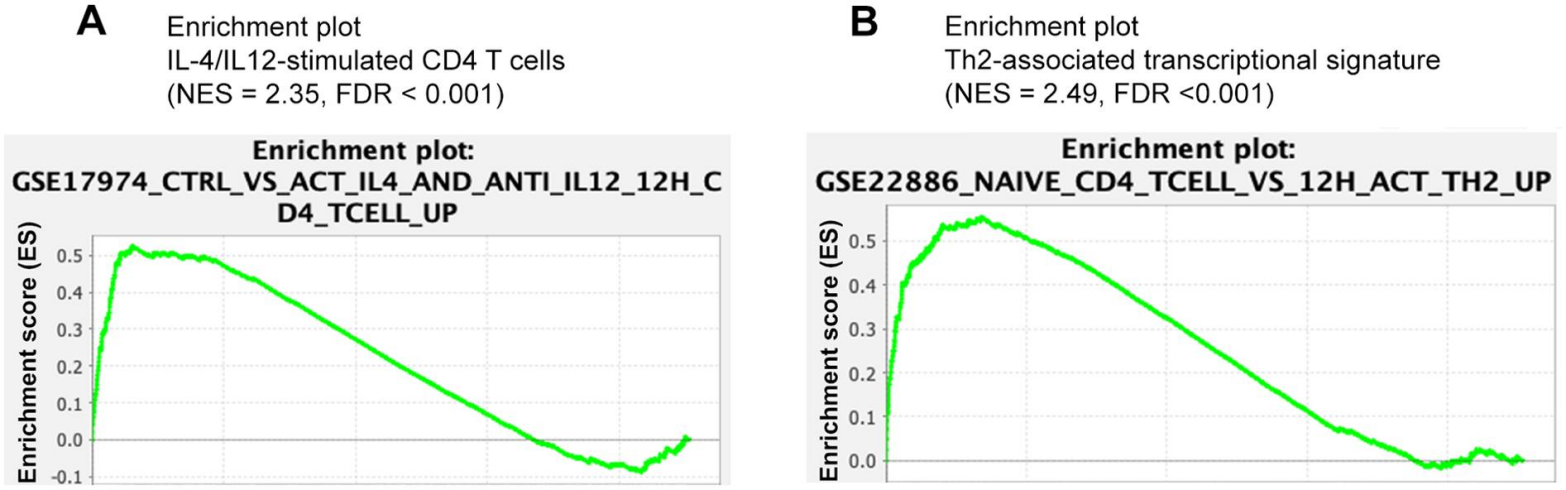
Gene Set enrichment analysis of peripheral blood T-cell DEGs. Enrichment plots showing significant positive enrichment of MSigDB immunologic signature gene sets in peripheral blood T-cell DEGs from AD patients. The enriched gene sets correspond to **(A)**, genes upregulated in CD4T cells stimulated with IL-4 and anti-IL-12 for 12 h and, **(B)** in activated Th2 cells compared with naïve CD4T cells.Positive enrichment indicates that genes from these signatures are overrepresented toward the top of the ranked gene list (24, 25).

Functional enrichment analysis of peripheral blood T-cell DEGs identified fewer canonical pathways by IPA (Supplementary Table 4). However, Gene Set Enrichment Analysis (GSEA) revealed significant positive enrichment of immune-related gene sets, including immunologic signatures related to IL-4–associated CD4T-cell responses and Th2 cell states. In particular, immunologic signature analysis showed positive enrichment of gene sets representing genes upregulated in IL-4– and IL-12–stimulated CD4T cells, as well as in activated Th2 lymphocytes (Figure 1).

**Figure 2:**
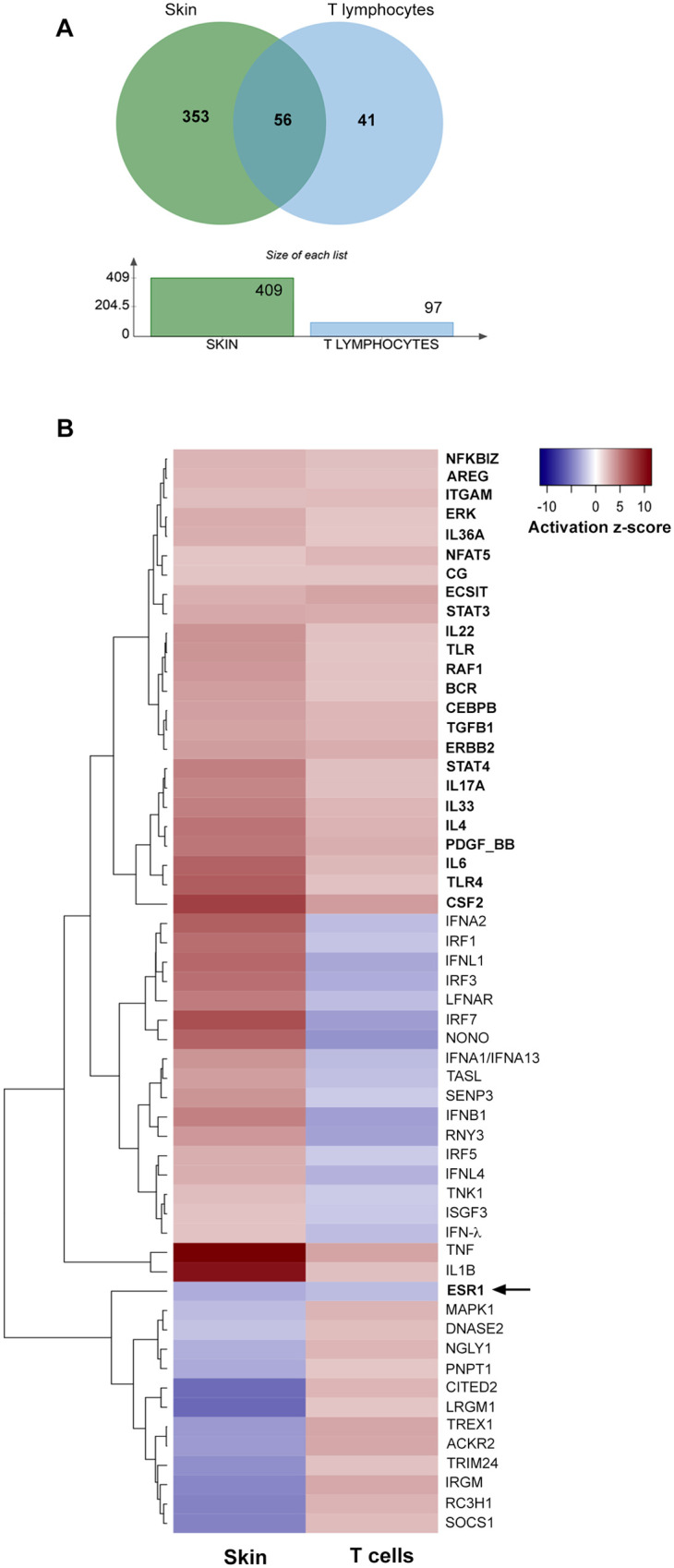
Shared upstream regulators in AD skin and peripheral blood T cells identified by IPA. **(A)** Venn diagram showing the number of upstream regulators predicted by Ingenuity Pathway Analysis (IPA) with an absolute activation *z*-score ≥ 2 in AD skin lesions and peripheral blood T lymphocytes. **(B)** Heatmap of the 56 upstream regulators common to both compartments. Colors represent the predicted activation state based on IPA activation *z*-scores, ranging from blue (negative *z*-scores; predicted inhibition) to red (positive *z*-scores; predicted activation). Notably, 24 regulators (bold) including several key inflammatory mediators (i.e., IL-4, IL-17A, IL-22, IL-33, and AREG) were consistently predicted to be activated, whereas ESR1 (see arrow) was the only regulator predicted to be inhibited across both compartments.

IPA results also describe potentially relevant regulators by detecting target gene over-representation for a variety of regulatory molecules (transcription regulators, microRNAs, cytokines, enzymes, etc.) among the collection of differentially expressed genes that are the subject of the study. Increased or decreased activity of the regulator is also predicted and expressed as a *z*-score value. Upstream regulator analysis identified a substantial overlap in predicted regulatory molecules between skin and peripheral blood T cells. A total of 56 upstream regulators were shared between both compartments. Among these, 24 regulators—including AREG, IL-22, IL-17A, IL-4, IL-33 and IL-36A—were predicted to be activated in both skin and blood, whereas ESR1 was consistently predicted to be inhibited (Figure 2). The remaining shared regulators showed divergent predicted activity between compartments.

These findings indicate that, although transcriptional changes differ markedly between skin and peripheral blood at the individual gene level, both compartments exhibit a shared inflammatory regulatory landscape in atopic dermatitis.

### Network analysis identifies key hub genes and functional modules

To identify key genes and biological modules driving the transcriptional changes observed in atopic dermatitis, protein–protein interaction (PPI) networks were constructed from differentially expressed genes in skin and peripheral blood T cells.

The PPI network derived from skin DEGs comprised 1,023 nodes and 3,507 edges. Hub genes were identified using betweenness centrality as a measure of network connectivity. Several of the top-ranked hub genes were among the most highly upregulated genes in AD skin, including CXCL8, CXCL10, CXCL11, IL13, S100A7, ALOX15 and MMP3 (Table 1), highlighting their central role in cutaneous inflammation.

**Table 1:** Hub genes in skin PPI network among the 100 most overexpressed in AD biopsies.

Gene name	Description	LogFC
CXCL10	C-X-C motif chemokine ligand 10	3.35
CXCL8	C-X-C motif chemokine ligand 8	4.63
CXCL11	C-X-C motif chemokine ligand 11	2.98
IL13	Interleukin 13	3.97
S100A7	S100 calcium binding protein A7	7.03
SOX2	SRY-box transcription factor 2	2.57
NTRK1	Neurotrophic receptor tyrosine kinase 1	4.75
RGS1	REM2 and RAB-like small GTPase1	2.6
ALOX15	Arachinodate 15-lipoxygenase	3.63
SERPINE1	Serine protease inhibitor (SERPIN) family protein	2.87
GZMB	Granzyme B	3.87
PI3	Peptidase inhibitor 3	4.88
MMP3	Matriz Metallopeptidase 3	3.96

Genes are listed from highest to lowest betweenness centrality index.

**Figure 3:**
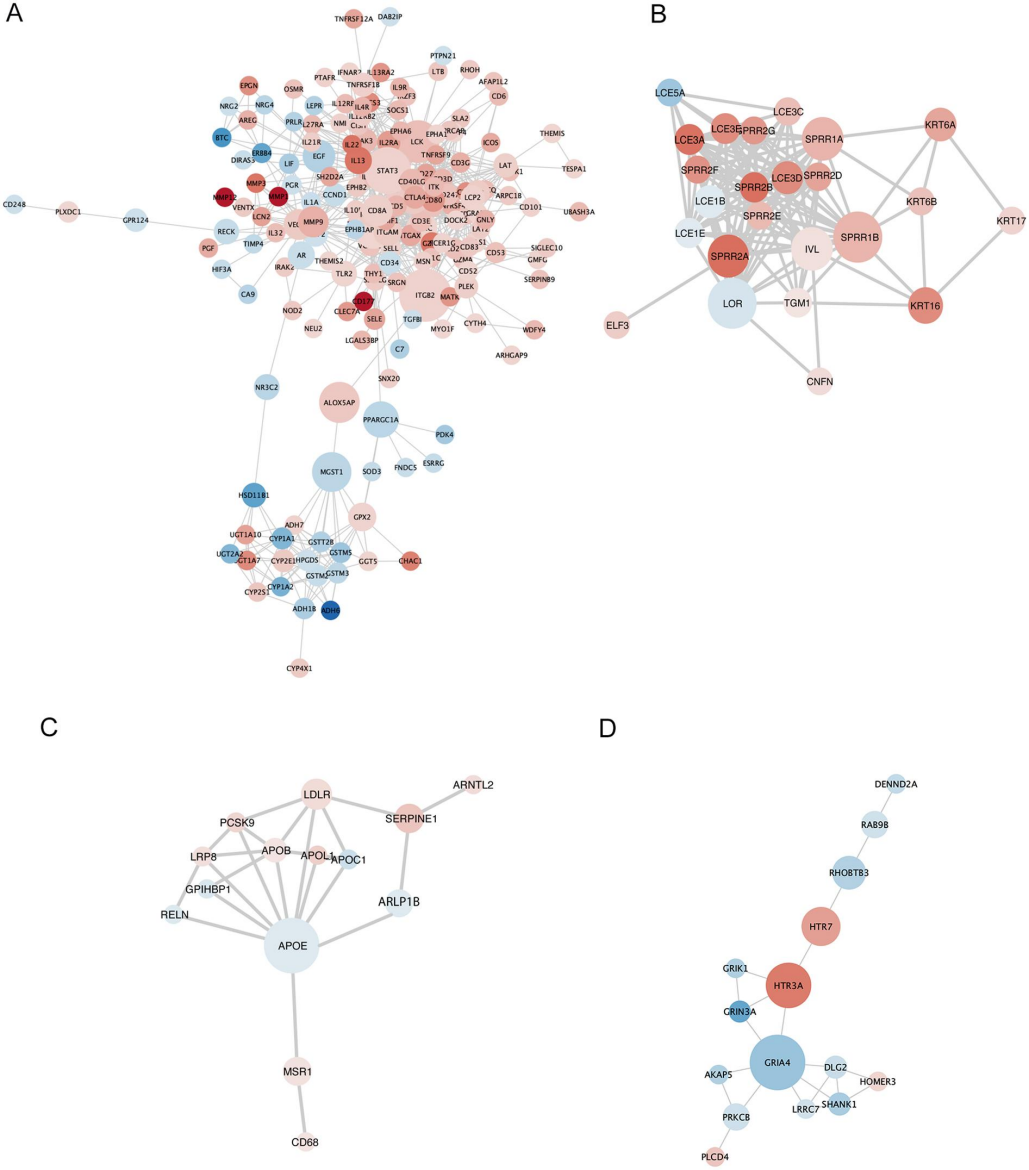
Functional clusters from skin PPI network. PPI network was constructed from DEG from AD skin and subjected to clustering analysis in Cytoscape, using the MCL (Markov cluster) algorithm implemented in the ClusterMaker plugin. **(A)** Cluster associated with immune system processes. **(B)** cluster associated with keratinization, **(C)** cluster associated with lipid metabolism and **(D)** cluster associated with transmission of nerve impulse and glutamate signaling pathway. Node size is associated with Betweenness Centrality score, red nodes correspond to upregulated genes (LogFc > 1), and blue nodes to downregulated genes (LogFc < 1); color intensity increases as LogFc.

Clustering analysis of the skin PPI network identified distinct functional modules. As expected, the largest cluster was associated with immune system processes, with ITGB2, STAT3 and LCK emerging as central nodes (Figure 3A). Additional clusters were associated with keratinization, lipid metabolism, and neuro-immune signaling pathways related to transmission of nerve impulse and glutamate signaling (Figures 3B–D). Within this latter cluster, GRIA4 and HTR3A showed the highest centrality scores, suggesting a potential role for glutamatergic signaling in AD-associated sensory and inflammatory responses.

The PPI network derived from peripheral blood T cell DEGs was smaller, comprising 103 nodes and 203 edges. The top hub genes included KLRC1, TNFRSF10A, IL1B, OSM and ALAS2, and functional modules were mainly related to T cell activation, differentiation and chemotaxis. Only four hub genes—CXCL8, CCL2, CA2 and TK1—were shared between skin and peripheral blood networks, underscoring the compartment-specific organization of gene networks in AD.

### Common differentially expressed genes across compartments identify candidate biomarkers

**Figure 4:**
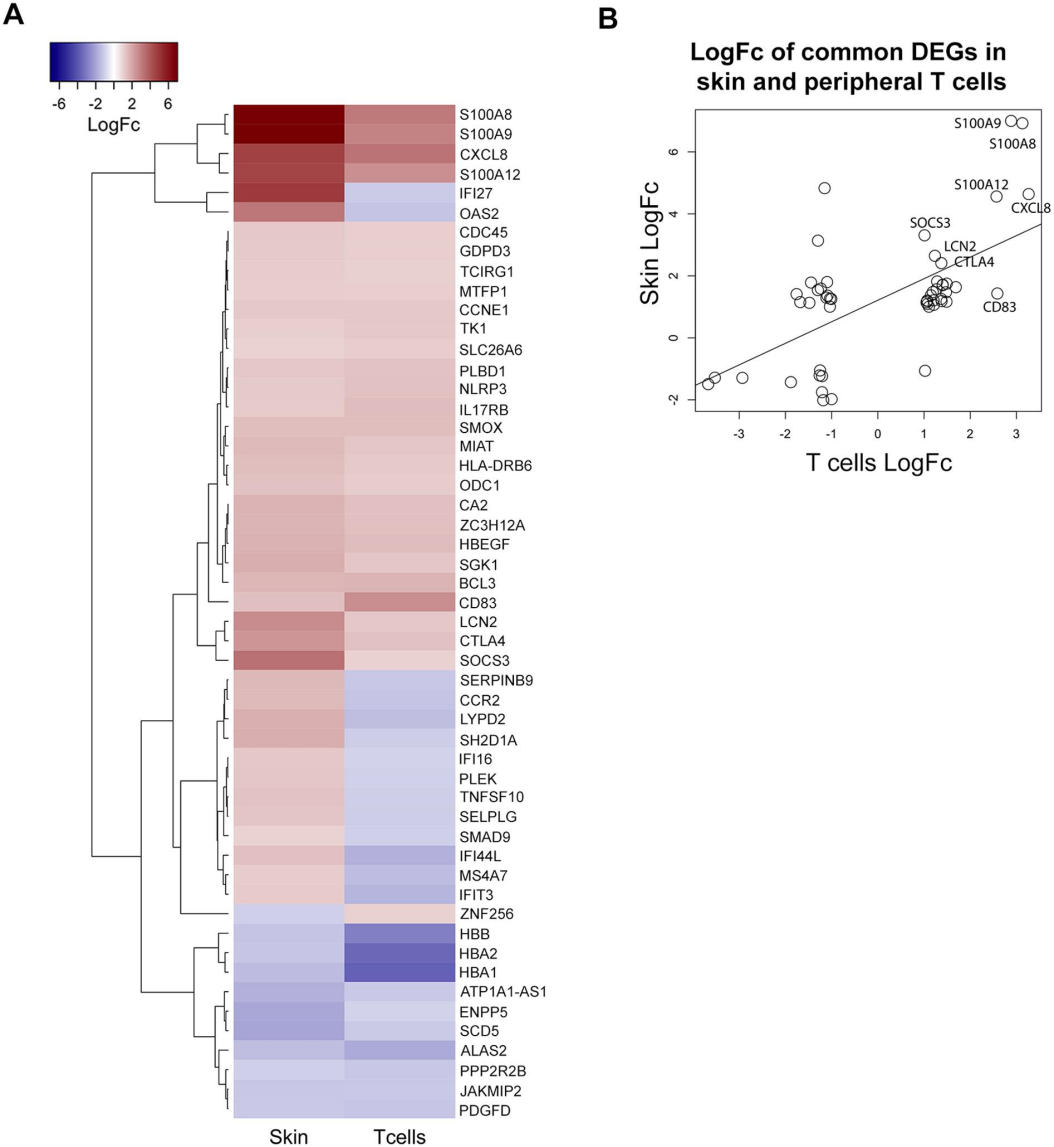
Common DEGs in skin and peripheral T cells of AD. **(A)** Heatmap of common DEGs in skin and peripheral T cells; heatmap colors range from blue to red, for negative and positive LogFc values, respectively. **(B)** Scatter plot showing the correlation of LogFc values of 52 common DEGs in skin and peripheral T cells.

To identify candidate molecules with potential relevance for systemic disease monitoring, we focused on genes that were differentially expressed in both lesional skin and peripheral blood T cells. Fifty-two DEGs were shared between both compartments, of which 37 showed concordant direction of expression (either upregulated or downregulated) in skin and blood (Figure 4A).

Among these shared genes, we prioritized those most likely to represent robust and biologically meaningful signals across compartments. Therefore, among the shared DEGs, we specifically selected genes showing the largest magnitude of expression change (logFC) in both AD skin lesions and peripheral blood T cells. This approach ensures that selected candidates reflect strong and consistent transcriptional alterations in both tissue-specific and systemic contexts, as illustrated in Figure 4B. Based on these criteria, seven candidate molecules were selected for further evaluation in serum samples: S100A9, S100A8, CXCL8, CD83, CTLA4, LCN2 and SOCS3.

This strategy allowed the selection of candidate biomarkers grounded in both tissue-specific and systemic transcriptional alterations, rather than isolated changes in a single compartment.

### Serum validation of candidate biomarkers and association with disease severity

To assess the clinical relevance of selected candidate molecules, their serum levels were analyzed in an independent cohort of 15 patients with moderate-to-severe atopic dermatitis prior to initiation of systemic treatment. Patients had a mean Eczema Area and Severity Index (EASI) score of 25.7 (range: 6.6–42.0). Clinic characteristics of patients are shown in Table 2.

**Table 2:** Phenotypic characteristics of patients.

Patient	Sex*	Age (y)	Smoking	Disease duration (y)	EASI
1	F	56	Yes	40	15.5
2	M	29	No	19	32
3	M	25	No	4	36.4
4	F	40	No	40	6.6
5	M	29	No	29	42
6	M	32	Yes	10	25.5
7	F	37	No	37	12.6
8	M	41	No	38	34.8
9	M	43	Yes	39	10.4
10	F	55	No	54	32.2
11	M	23	No	23	39.2
12	F	27	No	27	36
13	F	58	No	20	12.6
14	M	36	No	21	32
15	F	18	No	18	18.4

* F, female; M, male.

**Figure 5:**
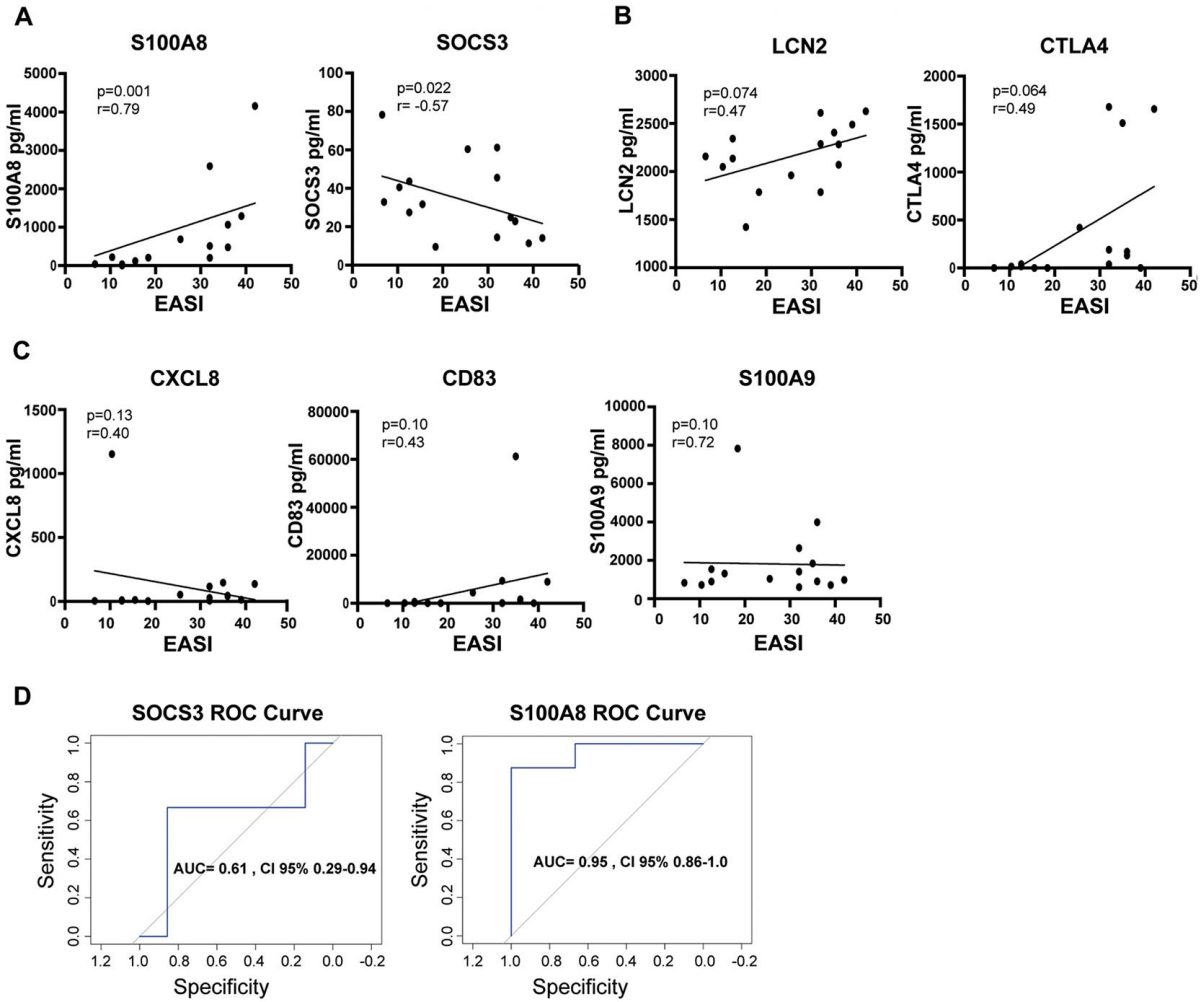
Candidate biomarkers and their association with disease severity in AD. **(A–C)** Correlation of serum protein levels (pg/mL), measured by multiplex antibody array and ELISA, with Eczema Area and Severity Index (EASI) scores in patients with AD (*n* = 15), analyzed using Spearman's rank correlation test. **(A)** SOCS3 and S100A8, showing significant correlations with disease severity. **(B)** LCN2 and CTLA4, showing trends toward correlation. **(C)** CXCL8, CD83, and S100A9, showing no significant association with EASI. **(D)** Receiver operating characteristic (ROC) curves for S100A8 and SOCS3 to discriminate disease severity (EASI > 21). Area under the curve (AUC) values with 95% confidence intervals (CI) are shown.

The circulating levels of cytokines were quantified in all patients. Median (IQR) levels were as follows: S100A9: 1,020 (847–1,763) pg/mL, S100A8: 346.9 (98.48–1,119) pg/mL, CXCL8: 27.7 (5.9–116.4) pg/mL, CD83: 124 (13.2–4,414) pg/mL, CTLA4: 41.50 (ND-420) pg/mL, LCN2: 2,157 (1,960–2,406) pg/mL, and SOCS3: 27.46 (14.50–45.53). Serum analysis revealed a significant positive correlation between disease severity and S100A8 levels (Spearman *r* = 0.79, *p* = 0.001), as well as a significant negative correlation between EASI score and SOCS3 levels (*r* = –0.57, *p* = 0.02) (Figure 5A). In addition, positive trends toward association with disease severity were observed for LCN2 (*r* = 0.47, *p* = 0.074) and CTLA4 (*r* = 0.49, *p* = 0.064) (Figure 5B), as well as CXCL8, CD83 and S100A9 (Figure 5C) although these did not reach statistical significance. ROC curve analysis using a binary definition of disease severity (EASI >21) showed that SOCS3 had a limited discriminatory capacity (AUC = 0.62, 95% CI: 0.30–0.94), with an optimal threshold of 26.18 yielding a sensitivity of 66.7% and a specificity of 85.7% (Figure 5D right). In contrast, S100A8 demonstrated a higher discriminatory performance (AUC = 0.96, 95% CI: 0.86–1.00), with an optimal cut-off of 346.85, corresponding to a sensitivity of 87.5% and a specificity of 100% (Figure 5D left).

These results support the relevance of circulating S100A8 and SOCS3 as biomarkers reflecting disease activity in atopic dermatitis and suggest that additional molecules identified through integrative transcriptomic analyses may contribute to systemic signatures of disease severity.

## Discussion

In this study, we performed an integrative transcriptomic analysis of lesional skin and peripheral blood T lymphocytes to identify molecular programs shared across compartments in atopic dermatitis and to explore their potential relevance as circulating biomarkers of disease activity. By combining differential gene expression, pathway enrichment, upstream regulator prediction and network-based analyses, we identified a set of molecules and regulatory pathways that link local cutaneous inflammation with systemic immune alterations.

One of the main findings of this work is that, despite the limited overlap of individual differentially expressed genes between skin and peripheral blood T cells, both compartments share a common inflammatory regulatory landscape. While transcriptional changes were more pronounced in lesional skin, reflecting tissue-specific inflammation, upstream regulator analyses revealed convergence at the level of cytokine-driven pathways, including IL-4, IL-17A, IL-22, IL-33 and AREG signaling. These results support the concept that systemic immune alterations in AD may not be adequately captured by direct gene-by-gene comparisons, but rather by higher-order regulatory programs that orchestrate inflammatory responses across tissues.

Network analysis further highlighted key hub genes and functional modules relevant to AD pathophysiology. In skin, immune-related clusters predominated, as expected, but additional modules related to keratinization, lipid metabolism and neuro-immune signaling were also identified. Of particular interest, a cluster associated with glutamate signaling and transmission of nerve impulse emerged, with GRIA4 identified as a central hub gene. GRIA4 encodes an AMPA-type glutamate receptor subunit, and altered expression of glutamate receptors has been previously linked to sensory abnormalities and pruritus in inflammatory skin diseases (12). Given that itch and sensory hypersensitivity are hallmark features of AD, the identification of a neuro-immune module supports the growing recognition of neuronal pathways as contributors to disease pathogenesis and symptom generation.

A key objective of this study was to identify candidate circulating biomarkers that mirror cutaneous inflammation. By focusing on genes consistently dysregulated in both skin and peripheral blood T cells, we prioritized molecules with potential systemic relevance. Among these, S100A8 and SOCS3 emerged as the most robustly associated with disease severity at the protein level. Serum S100A8 levels showed a strong positive correlation with EASI score, in line with previous reports supporting its role as a marker of inflammatory activity in AD (13). In contrast, SOCS3 levels correlated negatively with disease severity, suggesting a potential protective or regulatory role.

SOCS3 is a well-known negative regulator of cytokine signaling, particularly within the JAK/STAT pathway, and has been implicated in the modulation of Th2 responses (14). Although increased SOCS3 expression has been reported in both skin and peripheral blood cells of patients with AD (15, 16), our upstream regulator analysis predicted reduced SOCS3 activity in lesional skin. The inverse association between serum SOCS3 levels and disease severity observed in our cohort supports this prediction and suggests that impaired SOCS3 function, rather than absolute gene expression levels, may contribute to unchecked inflammatory signaling in AD. However, the present study was not designed to address the mechanistic basis of these associations, and it remains to be determined whether these molecules play a causal role in disease pathogenesis or reflect downstream consequences of inflammatory signaling. Further mechanistic studies will be required to clarify their functional contribution.

In addition to S100A8 and SOCS3, LCN2 and CTLA4 showed trends toward positive association with disease severity. Although these correlations did not reach statistical significance, likely due to the limited sample size, both molecules have established roles in immune regulation and inflammation. LCN2 has been linked to neutrophil activation and epithelial stress responses, while CTLA4 reflects immune checkpoint regulation and T cell activation status. These findings warrant further investigation in larger cohorts to clarify their potential utility as biomarkers.

Beyond biomarker identification, our analysis also highlighted regulatory molecules that are relatively underexplored in AD. ESR1 was consistently predicted to be inhibited in both skin and peripheral blood T cells. Reduced ESR1 activity has been implicated in other inflammatory skin diseases (17), and recent experimental data suggest that restoring ESR1 signaling may have therapeutic benefit (18). Similarly, regulators such as INSIG1, IKZF2 (HELIOS) and CITED2, predicted to be inhibited in AD skin, are involved in lipid metabolism, regulatory T cell stability and hypoxia-related inflammatory responses, respectively (19–23). Dysregulation of these pathways may contribute to key features of AD, including epidermal hyperplasia, impaired immune regulation and skewed T cell responses.

This study has limitations. The transcriptomic analyses relied on publicly available datasets generated using different platforms and experimental designs, and the peripheral blood dataset was derived from pooled samples, precluding statistical testing at the individual level. In addition, the serum validation cohort was relatively small. Nevertheless, the consistency of the regulatory patterns observed across compartments and the validation of selected candidates at the protein level support the robustness of our integrative approach. Furthermore, although the selected candidate biomarkers were consistently dysregulated at the transcriptomic level in both skin and blood, protein expression was only assessed in serum samples. Additional validation at the tissue level, for example by immunohistochemistry, would provide further insight into the cellular sources and spatial distribution of these molecules.

It should also be noted that the biomarker selection strategy prioritized genes showing the largest magnitude of expression change across both compartments and known relevance to immune regulation, rather than including all concordant differentially expressed genes. While this approach was designed to enhance robustness and translational potential, it may have excluded additional candidates with more modest expression changes.

In conclusion, our findings demonstrate that atopic dermatitis is characterized by shared molecular regulatory programs across skin and peripheral blood, despite compartment-specific gene expression patterns. Circulating levels of S100A8 and SOCS3, in particular, reflect disease activity and may serve as accessible biomarkers for monitoring AD severity. More broadly, this study underscores the value of integrative skin–blood analyses to uncover systemic components of cutaneous inflammation and to identify translational targets relevant to patient management.

Integrative analysis of skin and peripheral blood transcriptomes reveals shared inflammatory regulatory programs in atopic dermatitis and identifies circulating biomarkers that reflect disease activity. In particular, serum levels of S100A8 and SOCS3 emerge as accessible indicators of cutaneous inflammation with potential utility for monitoring disease severity.

## Data Availability

The original contributions presented in the study are included in the article/Supplementary Material, further inquiries can be directed to the corresponding author.

## References

[1] Weidinger S Beck LA Bieber T Kabashima K Irvine AD. Atopic dermatitis. Nat Rev Dis Primers. (2018) 4:1. 10.1038/s41572-018-0001-z 29930242

[2] Ahn K Kim BE Kim J Leung DY. Recent advances in atopic dermatitis. Curr Opin Immunol. (2020) 66:14–21. 10.1016/j.coi.2020.02.007 32299014 PMC7554175

[3] Fukushima-Nomura A Kawasaki H Yashiro K Obata S Tanese K Ebihara T. An unbiased tissue transcriptome analysis identifies potential markers for skin phenotypes and therapeutic responses in atopic dermatitis. Nat Commun. (2025) 16:4981. 10.1038/s41467-025-59340-x 40456762 PMC12130345

[4] Eapen AA Parameswaran S Forney C Edsall LE Miller D Donmez O. Epigenetic and transcriptional dysregulation in CD4+ T cells in patients with atopic dermatitis. PLoS Genet. (2022) 18:e1009973. 10.1371/journal.pgen.1009973 35576187 PMC9135339

[5] Wang Y Wu Y Gu C Wang S Yin H Zhu R. Peripheral blood mononuclear cell- transcriptome signatures of atopic dermatitis and prediction for the efficacy of dupilumab. J Dermatol Sci. (2023) 111:83–92. 10.1016/j.jdermsci.2023.06.002 37349237

[6] Brunner PM Suárez-Fariñas M He H Malik K Wen H-C Gonzalez J. The atopic dermatitis blood signature is characterized by increases in inflammatory and cardiovascular risk proteins. Sci Rep. (2017) 7:8707. 10.1038/s41598-017-09207-z 28821884 PMC5562859

[7] Noh JY Shin JU Kim JH Kim SH Kim B-M Kim YH. ZAG Regulates the skin barrier and immunity in atopic dermatitis. J Invest Dermatol. (2019) 139:1648–1657.e7. 10.1016/j.jid.2019.01.023 30738053

[8] Kim JE Lee J Huh YJ Kim K Chaparala V Krueger JG. Genomic profiling of the overlap phenotype between psoriasis and atopic dermatitis. J Invest Dermatol. (2024) 144:43–52.e6. 10.1016/j.jid.2023.06.194 37419444 PMC11060321

[9] Tsoi LC Rodriguez E Degenhardt F Baurecht H Wehkamp U Volks N. Atopic dermatitis is an IL-13-dominant disease with greater molecular heterogeneity compared to psoriasis. J Invest Dermatol. (2019) 139:1480–9. 10.1016/j.jid.2018.12.018 30641038 PMC6711380

[10] Martin M. Cutadapt removes adapter sequences from high-throughput sequencing reads. EMBnet.J. (2011) 17:3. 10.14806/ej.17.1.200

[11] Godec J Tan Y Liberzon A Tamayo P Bhattacharya S Butte AJ. Compendium of immune signatures identifies conserved and Species-specific biology in response to inflammation. Immunity. (2016) 44:194–206. 10.1016/j.immuni.2015.12.006 26795250 PMC5330663

[12] Cabañero D Irie T Celorrio M Trousdale C Owens DM Virley D. Identification of an epidermal keratinocyte AMPA glutamate receptor involved in dermatopathies associated with sensory abnormalities. Pain Rep. (2016) 1:e573. 10.1097/PR9.0000000000000573 28210712 PMC5305184

[13] Jin S Park CO Shin JU Noh JY Lee YS Lee NR. DAMP Molecules S100A9 and S100A8 activated by IL-17A and house-dust mites are increased in atopic dermatitis. Exp Dermatol. (2014) 23:938–41. 10.1111/exd.12563 25308296

[14] Haque SJ Harbor PC Williams BR. Identification of critical residues required for suppressor of cytokine signaling-specific regulation of interleukin-4 signaling. J Biol Chem. (2000) 275:26500–6. 10.1074/jbc.275.34.26500 10950967

[15] Arakawa S Hatano Y Katagiri K. Differential expression of mRNA for Th1 and Th2 cytokine-associated transcription factors and suppressors of cytokine signalling in peripheral blood mononuclear cells of patients with atopic dermatitis. Clin Exp Immunol. (2004) 135:505–10. 10.1111/j.1365-2249.2004.02405.x 15008986 PMC1808976

[16] Horiuchi Y Bae S-J Katayama I. Overexpression of the suppressor of cytokine signalling 3 (SOCS3) in severe atopic dermatitis. Clin Exp Dermatol. (2006) 31:100–4. 10.1111/j.1365-2230.2005.01979.x 16309496

[17] Adachi A Honda T Egawa G Kanameishi S Takimoto R Miyake T. Estradiol suppresses psoriatic inflammation in mice by regulating neutrophil and macrophage functions. J Allergy Clin Immunol. (2022) 150:909–919.e8. 10.1016/j.jaci.2022.03.028 35589416

[18] Lu X Kuai L Huang F Jiang J Song J Liu Y. Single-atom catalysts-based catalytic ROS clearance for efficient psoriasis treatment and relapse prevention via restoring ESR1. Nat Commun. (2023) 14:6767. 10.1038/s41467-023-42477-y 37880231 PMC10600197

[19] Evers BM Farooqi MS Shelton JM Richardson JA Goldstein JL Brown MS. Hair growth defects in insig-deficient mice caused by cholesterol precursor accumulation and reversed by simvastatin. J Invest Dermatol. (2010) 130:1237–48. 10.1038/jid.2009.442 20090767 PMC2929004

[20] Yu W-Q Ji N-F Gu C-J Wang Y-L Huang M Zhang M-S. Coexpression of Helios in Foxp3+ regulatory T cells and its role in human disease. Dis Markers. (2021) 2021:5574472. 10.1155/2021/5574472 34257746 PMC8245237

[21] Berlow RB Dyson HJ Wright PE. Hypersensitive termination of the hypoxic response by a disordered protein switch. Nature. (2017) 543:447–51. 10.1038/nature21705 28273070 PMC5375031

[22] Pong Ng H Kim G-D Ricky Chan E Dunwoodie SL Mahabeleshwar GH. CITED2 limits pathogenic inflammatory gene programs in myeloid cells. FASEB J. (2020) 34:12100–13. 10.1096/fj.202000864R 32697413 PMC7496281

[23] Qin X Chen H Tu L Ma Y Liu N Zhang H. Potent inhibition of HIF1α and p300 interaction by a constrained peptide derived from CITED2. J Med Chem. (2021) 64:13693–703. 10.1021/acs.jmedchem.1c01043 34472840

[24] Subramanian A Tamayo P Mootha VK Mukherjee S Ebert BL Gillette MA. Gene set enrichment analysis: a knowledge-based approach for interpreting genome-wide expression profiles. Proc Natl Acad Sci. (2005) 102:15545–50. 10.1073/pnas.0506580102 16199517 PMC1239896

[25] Mootha VK Lindgren CM Eriksson K-F Subramanian A Sihag S Lehar J. PGC-1alpha-responsive genes involved in oxidative phosphorylation are coordinately downregulated in human diabetes. Nat Genet. (2003) 34:267–73. 10.1038/ng1180 12808457

