# Supplementary material for: Integrative skin–blood transcriptomic analysis identifies circulating biomarkers reflecting disease activity in atopic dermatitis
Source: Front Allergy. 2026 Jun 9;7:1837776. doi: 10.3389/falgy.2026.1837776 (PMC13286974; doi:10.3389/falgy.2026.1837776)
Supplement: Supplementary file 2 [file table2.docx]

Supplementary Table 2. DEG in peripheral blood T cells of AD vs Controls.

| **Gene symbol** | **logFC** |
| --- | --- |
| S100A8 | 3,128 |
| S100A9 | 2,884 |
| IL1B | 2,812 |
| S100A12 | 2,569 |
| CD83 | 2,544 |
| ARHGEF10 | 2,476 |
| CCDC58 | 2,367 |
| CPT1B | 2,288 |
| AHSA2 | 2,213 |
| PGGHG | 2,183 |
| CD14 | 2,062 |
| NRGN | 1,962 |
| CXCL8 | 1,941 |
| XIST | 1,918 |
| NR4A2 | 1,701 |
| BCL3 | 1,687 |
| HLA-DQB1 | 1,685 |
| AMT | 1,666 |
| JMJD7-PLA2G4B | 1,657 |
| GP9 | 1,621 |
| CABP5 | 1,609 |
| GNG11 | 1,605 |
| LOC644936 | 1,595 |
| LINC00926 | 1,580 |
| F13A1 | 1,573 |
| MST1 | 1,542 |
| CLDN5 | 1,537 |
| TREML1 | 1,514 |
| SDPR | 1,511 |
| ACRBP | 1,507 |
| GRASP | 1,501 |
| ARHGAP33 | 1,500 |
| HBEGF | 1,491 |
| IL17RB | 1,486 |
| NPIPB5 | 1,483 |
| RGL2 | 1,479 |
| CNTNAP1 | 1,473 |
| SIK1 | 1,470 |
| KIFC2 | 1,469 |
| SNORD68 | 1,469 |
| NAT8B | 1,454 |
| APBB3 | 1,453 |
| PER1 | 1,449 |
| RBM38 | 1,448 |
| DNAH1 | 1,440 |
| AVPI1 | 1,413 |
| MEGF6 | 1,407 |
| PPP1R35 | 1,405 |
| ZC3H12A | 1,402 |
| ATG16L2 | 1,387 |
| CTSK | 1,385 |
| MAP3K7CL | 1,376 |
| HEXDC | 1,376 |
| NPIPA1 | 1,371 |
| PLBD1 | 1,370 |
| SNHG9 | 1,347 |
| PELI1 | 1,347 |
| RHOT2 | 1,346 |
| NKTR | 1,344 |
| LY6G6F | 1,343 |
| VPS9D1 | 1,330 |
| CCL3 | 1,320 |
| HIST2H2BE | 1,309 |
| PCED1A | 1,304 |
| TMEM158 | 1,302 |
| CCDC84 | 1,286 |
| LRP5L | 1,276 |
| MIAT | 1,272 |
| SNORD36A | 1,267 |
| VCAN | 1,266 |
| HIST2H2AA4 | 1,258 |
| PPBP | 1,251 |
| PRC1 | 1,242 |
| TMEM119 | 1,241 |
| MCEMP1 | 1,240 |
| HRASLS | 1,239 |
| SMG1P1 | 1,238 |
| LCN2 | 1,235 |
| PNMA3 | 1,222 |
| CLASRP | 1,219 |
| TK1 | 1,211 |
| CA2 | 1,208 |
| HSH2D | 1,204 |
|  |  |
| SLC7A5 | 1,201 |
| MYH3 | 1,200 |
| CXorf65 | 1,199 |
| LAMA5 | 1,194 |
| LOC102725126///FRG1HP | 1,186 |
| TUBB1 | 1,179 |
| GINS2 | 1,178 |
| ENC1 | 1,174 |
| INTS6-AS1 | 1,172 |
| MAN2C1 | 1,169 |
| CD19 | 1,154 |
| OSER1 | 1,152 |
| PLCH2 | 1,149 |
| ODC1 | 1,148 |
| CENPT | 1,145 |
| AQP10 | 1,139 |
| PIDD1 | 1,138 |
| NPEPL1 | 1,136 |
| CAPN3 | 1,135 |
| OVGP1 | 1,134 |
| PAN2 | 1,126 |
| TYMS | 1,126 |
| SPATA20 | 1,122 |
| IRX3 | 1,120 |
| MDGA1 | 1,117 |
| NSUN5P1 | 1,115 |
| CLEC1B | 1,113 |
| G0S2 | 1,111 |
| OSM | 1,110 |
| CAPG | 1,106 |
| ADHFE1 | 1,103 |
| BRICD5 | 1,100 |
| PVALB | 1,098 |
| ARHGAP4 | 1,097 |
| SPACA6 | 1,096 |
| TMEM91 | 1,096 |
| ZNF692 | 1,095 |
| ITGA2B | 1,094 |
| KANSL2 | 1,089 |
| TSPAN9 | 1,088 |
| NOXA1 | 1,084 |
| MC1R | 1,083 |
| MXD3 | 1,082 |
| SNHG17 | 1,079 |
| CRIPAK | 1,077 |
| HOXB2 | 1,075 |
| SPOCD1 | 1,074 |
| SNHG1 | 1,072 |
| CDC45 | 1,069 |
| CCR10 | 1,064 |
| TFAM | 1,063 |
| ANXA3 | 1,063 |
| LMNA | 1,062 |
| RNASE2 | 1,050 |
| TMEM55B | 1,048 |
| CEP95 | 1,047 |
| HEMK1 | 1,040 |
| SNN | 1,040 |
| ACCS | 1,035 |
| PPIEL | 1,030 |
| ASGR1 | 1,030 |
| ZNF256 | 1,022 |
| CAPRIN2 | 1,020 |
| TEPSIN | 1,019 |
| NUDT14 | 1,017 |
| MPL | 1,014 |
| SNHG11 | 1,004 |
| GUSBP11 | 1,004 |
| IER3 | 1,004 |
| ENPP5 | -1,000 |
| AQR | -1,001 |
| CCDC102A | -1,004 |
| ZFY | -1,005 |
| TARP | -1,006 |
| IFI16 | -1,007 |
| SMARCA5 | -1,011 |
| ADRB2 | -1,011 |
| ATP6V1D | -1,012 |
| TCF12 | -1,015 |
| TMEM171 | -1,021 |
| RAP2A | -1,032 |
| ATP7A | -1,034 |
| PLEK | -1,035 |
| LRRC25 | -1,035 |
| ABCB1 | -1,035 |
| SAMD9 | -1,037 |
| C9orf64 | -1,038 |
| SNORD12C | -1,038 |
| ACSF3 | -1,039 |
| VPS37A | -1,043 |
| FAM118A | -1,046 |
| PDGFRB | -1,048 |
| KLHL9 | -1,050 |
| TLR7 | -1,050 |
| KLRC1 | -1,050 |
| CLEC2D | -1,050 |
| TTPAL | -1,051 |
| SUGT1 | -1,053 |
| EOMES | -1,054 |
| CYBB | -1,056 |
| TM2D2 | -1,057 |
| PTGER2 | -1,062 |
| HDAC2 | -1,066 |
| GIMAP4 | -1,070 |
| ECHDC1 | -1,071 |
| NRCAM | -1,074 |
| NAPEPLD | -1,075 |
| ACO1 | -1,077 |
| MYOF | -1,077 |
| EIF1AY | -1,080 |
| GBP1 | -1,084 |
| TOR1AIP2 | -1,087 |
| TNFSF10 | -1,088 |
| ZNF189 | -1,094 |
| SH2D1A | -1,098 |
| ZNF260 | -1,107 |
| ABI3 | -1,108 |
| SELPLG | -1,112 |
| YIPF5 | -1,114 |
| PHF23 | -1,120 |
| TIGD7 | -1,124 |
| NEK7 | -1,126 |
| RNF20 | -1,128 |
| DCUN1D1 | -1,139 |
| ZNF613 | -1,144 |
| ARFIP1 | -1,146 |
| IFI27 | -1,152 |
| IFIT1 | -1,152 |
| IFNAR1 | -1,157 |
| RPE | -1,159 |
| ZMYM6 | -1,165 |
| CRIPT | -1,171 |
| C9orf69 | -1,181 |
| SCD5 | -1,181 |
| RAB10 | -1,189 |
| TM4SF19 | -1,191 |
| SLC39A8 | -1,192 |
| DHRS4-AS1 | -1,193 |
| FCRL3 | -1,196 |
| ENPP4 | -1,200 |
| JAKMIP2 | -1,207 |
| ATP1A1-AS1 | -1,209 |
| HSPA1A | -1,219 |
| GIN1 | -1,221 |
| CMTR2 | -1,222 |
| PAQR8 | -1,225 |
| MKKS | -1,230 |
| GIMAP1 | -1,232 |
| SERPINB9 | -1,233 |
| PPHLN1 | -1,236 |
| IGIP | -1,237 |
| DCAF12 | -1,238 |
| CENPQ | -1,242 |
| PPP3R1 | -1,246 |
| PALLD | -1,247 |
| PPP2R2B | -1,250 |
| G3BP1 | -1,255 |
| SLCO4C1 | -1,256 |
| PDGFD | -1,260 |
| TMEM35B | -1,274 |
| IRF2BPL | -1,281 |
| HRASLS2 | -1,281 |
| OAS2 | -1,295 |
| CCZ1B | -1,297 |
| CCR2 | -1,298 |
| ACKR3 | -1,307 |
| ADGRG1 | -1,323 |
| ZSWIM7 | -1,333 |
| LRRN3 | -1,333 |
| PTGDR | -1,335 |
| PREPL | -1,361 |
| RAB22A | -1,365 |
|  |  |
| DCAF7 | -1,371 |
| STT3A | -1,378 |
| SLC25A20 | -1,395 |
| CEPT1 | -1,419 |
| HELZ | -1,436 |
| LYPD2 | -1,446 |
| METTL13 | -1,450 |
| PRR5L | -1,463 |
| RNA18S5 | -1,476 |
| CMKLR1 | -1,479 |
| MS4A7 | -1,480 |
| KLRG1 | -1,530 |
| VPS35 | -1,560 |
| PRKAR1A | -1,572 |
| KLRC2 | -1,587 |
| CBX1 | -1,645 |
| SGK223 | -1,658 |
|  | -1,674 |
| IFIT3 | -1,679 |
| IFI44L | -1,758 |
| ALAS2 | -1,882 |
| ANGPTL6 | -1,903 |
| CX3CR1 | -2,158 |
| HBB | -2,931 |
| CDC42SE1 | -3,426 |
| HBA2 | -3,527 |
| HBA1 | -3,671 |
