# Supplementary material for: Integrative skin–blood transcriptomic analysis identifies circulating biomarkers reflecting disease activity in atopic dermatitis
Source: Front Allergy. 2026 Jun 9;7:1837776. doi: 10.3389/falgy.2026.1837776 (PMC13286974; doi:10.3389/falgy.2026.1837776)
Supplement: Supplementary file 3 [file table3.docx]

Supplementary Table 3. Canonical Pathways in AD skin

| **Ingenuity Canonical Pathways** | **B-H adjusted p-value** | **z-score** | **Predicted Activation State** |
| --- | --- | --- | --- |
| T Cell Recep Signaling | 3,54E-02 | 4,490 | Increased |
| FAK Signaling | 8,00E-12 | 4,386 | Increased |
| Osteoarthritis Pathway | 2,30E-03 | 4,271 | Increased |
| PKCθ Signaling in T Lymphocytes | 3,54E-02 | 4,025 | Increased |
| Dendritic Cell Maturation | 1,00E-04 | 3,889 | Increased |
| Phagosome Formation | 2,00E-07 | 3,878 | Increased |
| Crosstalk between Dendritic Cells and Natural Killer Cells | 1,90E-03 | 3,873 | Increased |
| TREM1 Signaling | 4,10E-03 | 3,873 | Increased |
| ICOS-ICOSL Signaling in T Helper Cells | 1,30E-03 | 3,638 | Increased |
| Th2 Pathway | 2,00E-06 | 3,528 | Increased |
| Leukocyte Extravasation Signaling | 1,90E-03 | 3,402 | Increased |
| IL-17 Signaling | 6,00E-08 | 3,162 | Increased |
| Th1 Pathway | 1,00E-04 | 2,985 | Increased |
| IL-15 Production | 3,23E-02 | 2,985 | Increased |
| Pulmonary Healing Signaling Pathway | 9,00E-04 | 2,920 | Increased |
| CD28 Signaling in T Helper Cells | 1,69E-02 | 2,840 | Increased |
| Wound Healing Signaling Pathway | 1,00E-04 | 2,832 | Increased |
| Role of IL-17A in Psoriasis | 3,00E-05 | 2,828 | Increased |
| MIF-mediated Glucocorticoid Regulation | 3,80E-02 | 2,828 | Increased |
| G-Protein Coupled Recep Signaling | 3,00E-03 | 2,778 | Increased |
| Interferon Signaling | 1,50E-03 | 2,714 | Increased |
| Type I Diabetes Mellitus Signaling | 1,77E-02 | 2,673 | Increased |
| Th17 Activation Pathway | 2,00E-07 | 2,668 | Increased |
| Cardiac Hypertrophy Signaling (Enhanced) | 1,30E-03 | 2,610 | Increased |
| Tumor Microenvironment Pathway | 6,00E-06 | 2,596 | Increased |
| Systemic Lupus Erythematosus In B Cell Signaling Pathway | 3,00E-05 | 2,546 | Increased |
| LPS/IL-1 Mediated Inhibition of RXR Function | 2,00E-06 | 2,333 | Increased |
| OX40 Signaling Pathway | 2,51E-02 | 2,333 | Increased |
| TEC Kinase Signaling | 1,40E-03 | 2,065 | Increased |
| Salvage Pathways of Pyrimidine Deoxyribonucleotides | 8,10E-03 | 2,000 | Increased |
| Inhibition of Matrix Metalloproteases | 3,70E-03 | -2,111 | Decreased |
| Antiproliferative Role of TOB in T Cell Signaling | 8,30E-03 | -2,121 | Decreased |
| Antioxidant Action of Vitamin C | 3,54E-02 | -2,324 | Decreased |
| Xenobiotic Metabolism CAR Signaling Pathway | 1,02E-02 | -2,414 | Decreased |
