# Supplementary material for: Integrative skin–blood transcriptomic analysis identifies circulating biomarkers reflecting disease activity in atopic dermatitis
Source: Front Allergy. 2026 Jun 9;7:1837776. doi: 10.3389/falgy.2026.1837776 (PMC13286974; doi:10.3389/falgy.2026.1837776)
Supplement: Supplementary file 4 [file table4.docx]

Supplementary Table 4. Peripheral blood T cells canonical pathways

| **Ingenuity Canonical Pathways** | **B-H adjusted p-value** | **z-score** | **Predicted Activation State** |
| --- | --- | --- | --- |
| Sirtuin Signaling Pathway | 1,41E-02 | 3,000 | Increased |
| Systemic Lupus Erythematosus In T Cell Signaling Pathway | 2,10E-03 | 2,197 | Increased |
| Dendritic Cell Maturation | 6,00E-04 | 2,121 | Increased |
| OX40 Signaling Pathway | 8,00E-04 | -2,000 | Decreased |
| IL-8 Signaling | 1,23E-02 | -2,043 | Decreased |
| IL-9 Signaling | 1,51E-02 | -2,121 | Decreased |
| Type I Diabetes Mellitus Signaling | 9,00E-05 | -2,324 | Decreased |
| Systemic Lupus Erythematosus In B Cell Signaling Pathway | 5,70E-03 | -2,343 | Decreased |
| fMLP Signaling in Neutrophils | 4,89E-02 | -2,357 | Decreased |
| Role of PKR in Interferon Induction and Antiviral Response | 1,51E-02 | -2,524 | Decreased |
| Erythropoietin Signaling Pathway | 2,10E-03 | -2,694 | Decreased |
| NUR77 Signaling in T Lymphocytes | 1,00E-04 | -3,464 | Decreased |
